# Tooth image segmentation and root canal measurement based on deep learning

**DOI:** 10.3389/fbioe.2025.1565403

**Published:** 2025-06-09

**Authors:** Ziqing Chen, Qi Liu, Jialei Wang, Nuo Ji, Yuhang Gong, Bo Gao

**Affiliations:** ^1^ School of Biomedical Engineering, Sichuan University, Chengdu, China; ^2^ National Clinical Research Center for Oral Diseases, West China Hospital of Stomatology, Sichuan University, Chengdu, China

**Keywords:** tooth instance segmentation, CBCT, root canal measurement, deep learning, Attention U-net, V-Net

## Abstract

**Indroduction:**

This study aims to develop a automated method for tooth segmentation and root canal measurement based on cone beam computed tomography (CBCT) images, providing objective, efficient, and accurate measurement results to guide and assist clinicians in root canal diagnosis grading, instrument selection, and preoperative planning.

**Methods:**

We utilizes Attention U-Net to recognize tooth descriptors, crops regions of interest (ROIs) based on the center of mass of these descriptors, and applies an integrated deep learning method for segmentation. The segmentation results are mapped back to the original coordinates and position-corrected, followed by automatic measurement and visualization of root canal lengths and angles.

**Results:**

Quantitative evaluation demonstrated a segmentation Dice coefficient of 96.33%, Jaccard coefficient of 92.94%, Hausdorff distance of 2.04 mm, and Average surface distance of 0.24 mm - all surpassing existing methods. The relative error of root canal length measurement was 3.42% (less than 5%), and the effect of auto-correction was recognized by clinicians.

**Discussion:**

The proposed segmentation method demonstrates favorable performance, with a relatively low relative error between automated and manual measurements, providing valuable reference for clinical applications.

## 1 Introduction

Endodontics and periapical diseases are common dental conditions, and root canal therapy is the most effective treatment. Determining the working length of the root canal is crucial for improving the success rate of the procedure. cone-beam computed tomography (CBCT), with its high spatial resolution, is ideal for 3D imaging (Cui et al., 2022). Dental models reconstructed by CBCT accurately present the patient’s 3D anatomical structure and dental morphology, which helps to design efficient and precise treatment plans (Wang et al., 2024), and are widely used in oral surgery and digital dentistry. Several studies (Abulhamael et al., 2024; Kumari et al., 2024; Izadi et al., 2024) have demonstrated that CBCT-based measurements of root canal length are both accurate and reliable when compared to the gold standard. Therefore, the segmentation of a single tooth from a CBCT image and its automatic measurement is crucial for endodontic treatment and digital dentistry.

Segmenting individual teeth from CBCT scans presents significant challenges due to factors such as tooth occlusion, similarities in the densities of teeth and alveolar bone, and the propensity for neighboring teeth to be misidentified (Zhang et al., 2024; Jang et al., 2022; Zhang et al., 2021). Traditional tooth segmentation techniques (Al-sherif et al., 2012; Hiew et al., 2010; Keustermans et al., 2012; Evain et al., 2017; Zichun et al., 2020; Jiang et al., 2022; Gan et al., 2018; Jiang et al., 2024; Wang et al., 2019)—including thresholding, graph-cutting, and level-set methods—are typically semi-automatic and exhibit limited robustness. These methods often encounter issues of under-segmentation or over-segmentation and are sensitive to noise artifacts.

Deep learning has been widely applied in teeth image segmentation, with its ability to detect subtle anatomical features and complex textures, have significantly improved the accuracy of CBCT dental image segmentation. Cui et al. (2019) employed a deep supervised model utilizing the proposed 3D region proposal network (RPN) for the segmentation of single teeth. Chung et al. (2020) achieved single tooth segmentation through pose regression and convolutional neural networks; however, they did not address the issue of overlapping voxels between neighboring teeth. Chen et al. (2020) introduced a method that combines a 3D full convolutional network with watershed transform to segment individual teeth, yet this approach encounters challenges when segmenting neighboring teeth with indistinct boundaries. Several studies (Shaheen et al., 2021; Wang et al., 2023; Gong et al., 2024; Tan et al., 2024; Wu et al., 2020; Cui et al., 2021) have successfully realized single tooth segmentation using multi-stage network segmentation methods.

Manual measurement is characterized by instability, dependence on the operator’s experience, and time consumption. In contrast, automatic measurement provides objective, convenient, and effective quantitative results. Currently, research on automatic tooth measurement is limited (Piasecki et al., 2018; Mourao et al., 2025), with the majority of studies relying on manual or semi-automatic methods that often focus on 2D measurements, which do not fully leverage 3D spatial information.

In this paper, we proposed a multi-stage, automated method for individual tooth segmentation and root canal measurement. The segmentation results were effective and robust, and the measurements were consistent with the physician’s estimates. These measurements can guide and support clinicians in root canal diagnosis grading, instrument selection, and preoperative planning, thereby offering significant clinical value.

## 2 Methods

**FIGURE 1:**
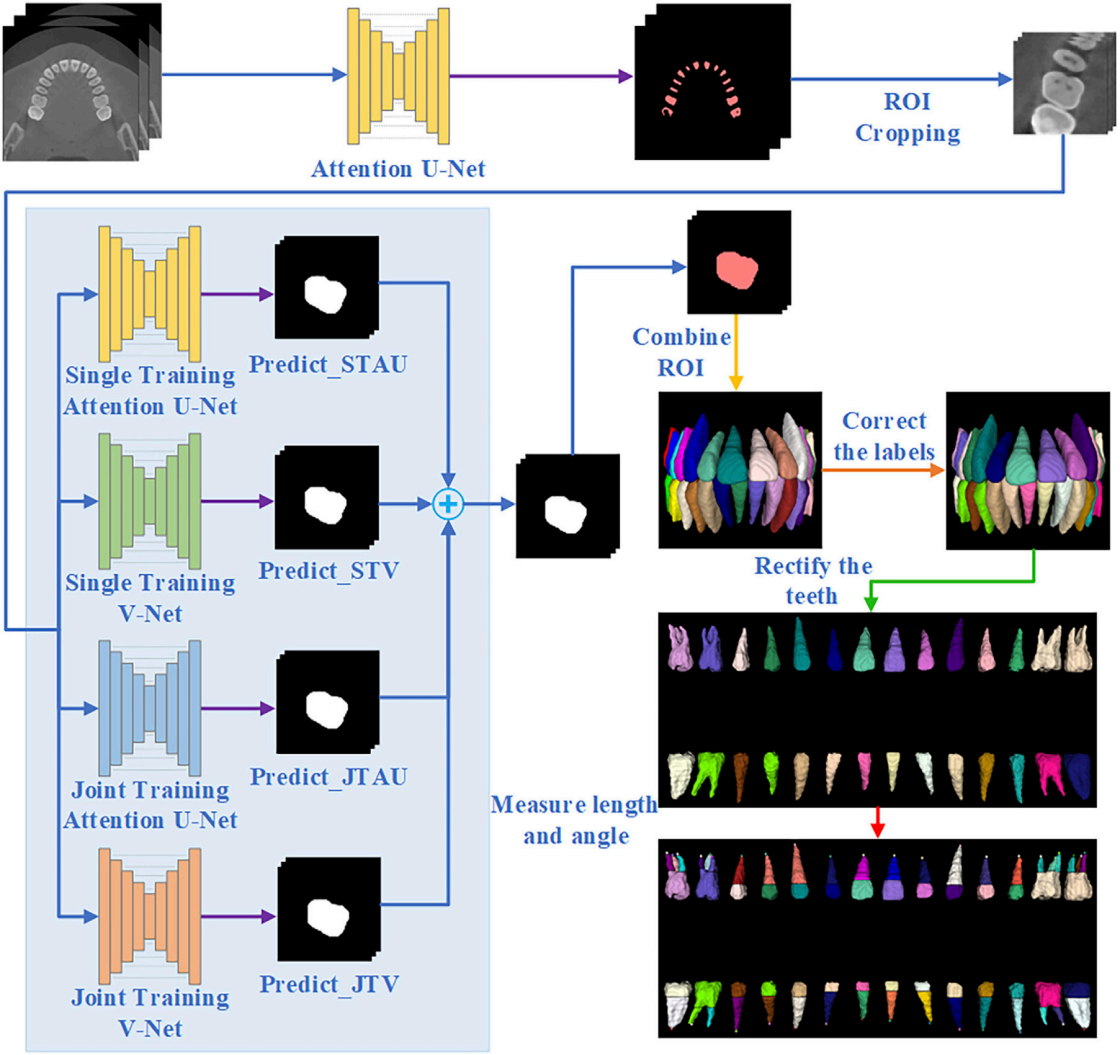
Overall flowchart of tooth segmentation and working length angle measurement.

The overall workflow of the proposed automated method for individual tooth segmentation and root canal measurement is illustrated in Figure 1.

### 2.1 Tooth detection

Due to the tooth structure and natural occlusion, adhesion often occurs between adjacent teeth and between upper and lower teeth in CBCT images. Thus, in this study, the remaining tooth portion with tooth boundaries removed is used as a descriptor for tooth localization, and tooth descriptor segmentation is performed by the Attention U-Net network. The Attention U-Net (Oktay et al., 2018) with an attention mechanism for descriptor segmentation, which allows the model to focus more on important regions of the image and reduce computation on irrelevant areas. In the training stage, the loss function used is Generalized Dice Focal Loss (GDFL), which combines the Generalized Dice loss and Focal loss, accounting for voxel overlap and the weighting of hard-to-classify samples.

### 2.2 Tooth segmentation

We take the center of mass of the tooth descriptors as the center of the tooth and crop out the tooth ROI of size (64,64,96). This ROI is used for individual tooth segmentation.

This study employs an ensemble learning algorithm to segment individual tooth, utilizing the integration of Attention U-Net and V-Net (Milletari et al., 2016). V-Net is specifically designed for processing three-dimensional volume data, thereby enhancing the capture of three-dimensional structural information. By integrating these two networks, we can leverage their respective advantages to reduce the incidence of false positives and false negatives, ultimately improving overall segmentation quality. Furthermore, the combination of the two networks’ characteristics enhances the model’s robustness against different image types and variations.

Initially, we train Attention U-Net (STAU) and V-Net (STV) separately using the same training set. Subsequently, we jointly train Attention U-Net (JTAU) and V-Net (JTV) with the same training set. The loss functions of both U-Net and V-Net are incorporated as a new loss function to guide the joint training of the models. Given the inherent randomness associated with training individual networks, we conduct ten training times for each network (i.e., training Attention U-Net alone ten times, training V-Net alone ten times, and jointly training the two networks ten times).

GDFL is employed as the loss function during the individual training phase of the network. In the joint training phase, the loss function is defined as follows (Equation 1):

$$Loss_{\mathrm{joint}}=Loss_{\mathrm{GDFL}}^{\mathrm{AUN}}+Loss_{\mathrm{GDFL}}^{VN}$$ (1)

Where $Loss_{\mathrm{GDFL}}^{\mathrm{AUN}}$ represents the GDFL of Attention U-Net prediction results, and $Loss_{\mathrm{GDFL}}^{VN}$ denotes the GDFL of V-Net prediction results.

In this study, we propose a new composite metric that integrates Dice coefficients, Hausdorff Distance (HD) coefficients, and Average Surface Distance (ASD) coefficients. Given that the Dice coefficient and the Jaccard coefficient are interchangeable, we have excluded the Jaccard coefficient from the calculation of the composite metric. The composite metric $C_{\mathrm{Metric}}$ is expressed as fallows (Equation 2):

$$C_{\mathrm{Metric}}=Dice+\frac{1}{HD}+\frac{0.1}{ASD}$$ (2)

The network segmentation effect is directly proportional to the Dice coefficient and inversely proportional to the HD coefficient and ASD coefficient. Consequently, our composite indicator $C_{\mathrm{Metric}}$ is directly proportional to the segmentation effect. The value of HD typically ranges from a few millimeters, while the value of ASD generally falls within a few tenths of a millimeter. To mitigate the excessive influence of the ASD value on $C_{\mathrm{Metric}}$ , we employ a coefficient of 0.1.

During the network testing phase, for a given test data set (i.e., a single tooth ROI), we obtain the training results from ten STAU instances and compute the $C_{\mathrm{Metric}}$ for these ten results. We then select the network result with the largest $C_{\mathrm{Metric}}$ as the STAU result, denoted as $pred_{\mathrm{STAU}}$ . Similarly, we derive the results for STV $\left(pred_{\mathrm{STV}}\right)$ , for JTAU $\left(pred_{\mathrm{JTAU}}\right)$ , and for JTV $\left(pred_{\mathrm{JTV}}\right)$ .

We employ the ensemble learning method to derive the final output result. This result is calculated as the weighted sum of the outputs from each network, where the weights are determined by their respective $C_{\mathrm{Metric}}$ values (Equation 3):

$$pred=\frac{C_{\mathrm{Metric}}^{\mathrm{STAU}}}{C_{\mathrm{Metric}}^{\mathrm{sum}}}pred_{\mathrm{STAU}}+\frac{C_{\mathrm{Metric}}^{\mathrm{STV}}}{C_{\mathrm{Metric}}^{\mathrm{sum}}}pred_{\mathrm{STV}}+\frac{C_{\mathrm{Metric}}^{\mathrm{JTAU}}}{C_{\mathrm{Metric}}^{\mathrm{sum}}}pred_{\mathrm{JTAU}}+\frac{C_{\mathrm{Metric}}^{\mathrm{JTV}}}{C_{\mathrm{Metric}}^{\mathrm{sum}}}pred_{\mathrm{JTV}}$$ (3)

Where. $C_{\mathrm{Metric}}^{\mathrm{sum}}=C_{\mathrm{Metric}}^{\mathrm{STAU}}+C_{\mathrm{Metric}}^{\mathrm{STV}}+C_{\mathrm{Metric}}^{\mathrm{JTAU}}+C_{\mathrm{Metric}}^{\mathrm{JTV}}$

### 2.3 Re-labeling and tooth position correction

The internationally recognized Fédération dentaire internationale (FDI) tooth position representation was utilized to label the teeth in this study. After obtaining the segmentation results for the ROIs, these results were mapped back to the original coordinates, followed by a correction of the tooth labels based on their relative positions.

Due to the curved arrangement of teeth in natural occlusion and the influence of the imaging angle, the crown surfaces of the teeth do not align with the slice plane. To address this discrepancy, tooth positional correction was implemented to minimize alignment-related measurement biases. Given the irregular morphology, variable positioning, and occasional absence of third molars (wisdom teeth), they were excluded from both positional correction and root canal measurement analyses.

For each tooth, the crown portion is intercepted, and correction reference points are selected based on the projected shape characteristics of the crowns of different types of teeth, and the teeth are corrected sequentially along the XYZ direction. The specific order of correction is as follows: Step 1: Correct the tooth position along the Z-axis twice. Step 2: Correct the tooth position along the X-axis. Step 3: Correct the tooth position along the Y-axis. Step 4: Arrange the corrected teeth in sequence.

In this study, three methods were developed to select the correction reference point. Method one involves calculating the hull points in projected coordinates, designating the projected center point as the origin, and dividing the hull points into four quadrants. The correction reference points is then identified as the point farthest from the center point in each quadrant. The second method also divides the hull points into quadrants, similar to the first method. For each quadrant, the maximum distances ( $D_{X}$ and $D_{Y}$ ) of the hull points from the center are calculated in two directions. The correction reference point is determined as the point that meets $\left|x_{i}-x_{0}\right|>0.5\;*\;D_{X}$ and $\left|y_{i}-y_{0}\right|>0.5\;*\;D_{Y}$ and is closest to the edge of the quadrant. Where $\left(x_{0},y_{0}\right)$ represents the center point, $\left(x_{i},y_{i}\right)$ denotes a specific convex hull point. The third method establishes the correction reference point by calculating the minimum bounding rectangle of the projected coordinates. Based on the type and position of the teeth, different methods are employed to obtain correction angle reference points at each step.

### 2.4 Measurement of tooth working length and root canal curvature

Tooth working length refers to the distance from the crown reference point to the point where root canal preparation and filling should terminate (Sharma and Arora, 2010). The tooth is divided into two parts: the crown and the root. In single-canal teeth, a fixed-length portion is designated as the crown, while in multiple-canal teeth, the root is determined using Connected Component Analysis. The automatic measurement method quantifies the direct distance from the crown to the root apex, and directly calculates the straight-line distance from the center point of the crown to the center point of the root apex.

Schneider’s method is the most widely utilized technique for measuring the angle of root canal curvature. This angle is defined as the angle between the vector originating from the starting point of the root canal to the starting point of root canal curvature, and the vector from the starting point of root canal curvature to the endpoint of the root canal. In this study, we refer to the Schneider method to calculate the root canal curvature, and the key is to find the starting point of root canal curvature. The coordinates of the center point of the second layer to the penultimate layer center point are sequentially taken as candidate points, referred to as point j. The center point of the previous slice layer of the candidate point is denoted as point i, while the center point of the next slice layer of the candidate point is designated as point k. The angle between the vector from point i to point j and the vector from point j to point k is calculated, with the candidate point exhibiting the largest angle identified as the center point of the root canal curvature layer.

## 3 Experiments and results

### 3.1 Dataset

This study collected 39 CBCT images from West China Hospital of Stomatology, Sichuan University. All images were obtained from patients in natural or closed occlusion. The scanning parameters were: tube voltage 85.0 kV, current 4.0 mA, exposure time 17.5 s, pixel spacing 0.25 mm, and slice thickness 1.00 mm. The CBCT images had a width and height of 565 pixels, with 101 slices. The field of view measured 141.25 × 141.25 × 101 mm^3^;. The images in this study were manually labeled by specialized physicians utilizing the ITK-SNAP platform. Model training was performed based on Torch 2.4, using an NVIDIA RTX 3060 GPU.

The data were divided into training, validation, and test sets, with the training and validation sets referred to as training data. In the tooth detection stage, 27 cases were randomly selected for the training set, 3 cases for the validation set, and 9 cases for the test set. In the tooth segmentation stage, 30 training cases yielded 871 single-tooth ROIs, from which 800 ROIs were randomly selected for the training set, 71 ROIs for the validation set, and 275 ROIs were obtained from nine test cases for the test set.

Preprocessing: The images were normalized to an isotropic resolution of 0.4 × 0.4 × 0.4 mm^3^; and cropped to 256 × 256 × 256 while preserving complete tooth information.

Tooth ROI Cropping: The images were cropped to a size of 64 × 64 × 96, using the centroid of the tooth descriptor obtained in the tooth detection stage as the cropping center (for the training and validation sets, the cropping center was the tooth centroid).

Adding perturbation: The centroids of the tooth descriptors predicted in tooth detection stage may deviate from the true tooth centroids. Inspired by Ref. 26, we introduced perturbations to the centroids in the training and validation sets to better approximate the real situation and improve model robustness. The centroids of the training data were perturbed with a 50% probability, with a random offset of −8 to eight voxels applied along each axis. The new centroid was then used as the ROI cropping center, while non-perturbed centroids retained their original positions as the cropping center.

### 3.2 Evaluation metrics

This study assessed the detection of tooth descriptors by utilizing the ASD, HD, and the voxel distance between the predicted and actual tooth centroid, referred to as Centroid Distance (CD). This study employs the Dice coefficient, Jaccard coefficient, ASD, and HD to assess the results of tooth segmentation.

The voxel distance between the predicted tooth descriptor centroid and true tooth centroid is (Equation 4):

$$CD_{i}={\sqrt{{\left(x_{1}-x_{2}\right)}^{2}+{\left(y_{1}-y_{2}\right)}^{2}+{\left(z_{1}-z_{2}\right)}^{2}}}_{i\in L}$$ (4)

where L is the set of teeth, $\left(x_{1},y_{1},z_{1}\right)$ is the predicted tooth descriptor centroid, and $\left(x_{2},y_{2},z_{2}\right)$ is the true tooth centroid.

### 3.3 Tooth detection

The objective of the tooth detection stage is to obtain tooth descriptors that accurately localize individual teeth, avoiding both one-to-many and many-to-one mappings, as well as preventing the detection of non-tooth regions as tooth descriptors. The Attention U-Net employed in this study effectively accomplishes this goal.

**FIGURE 2:**
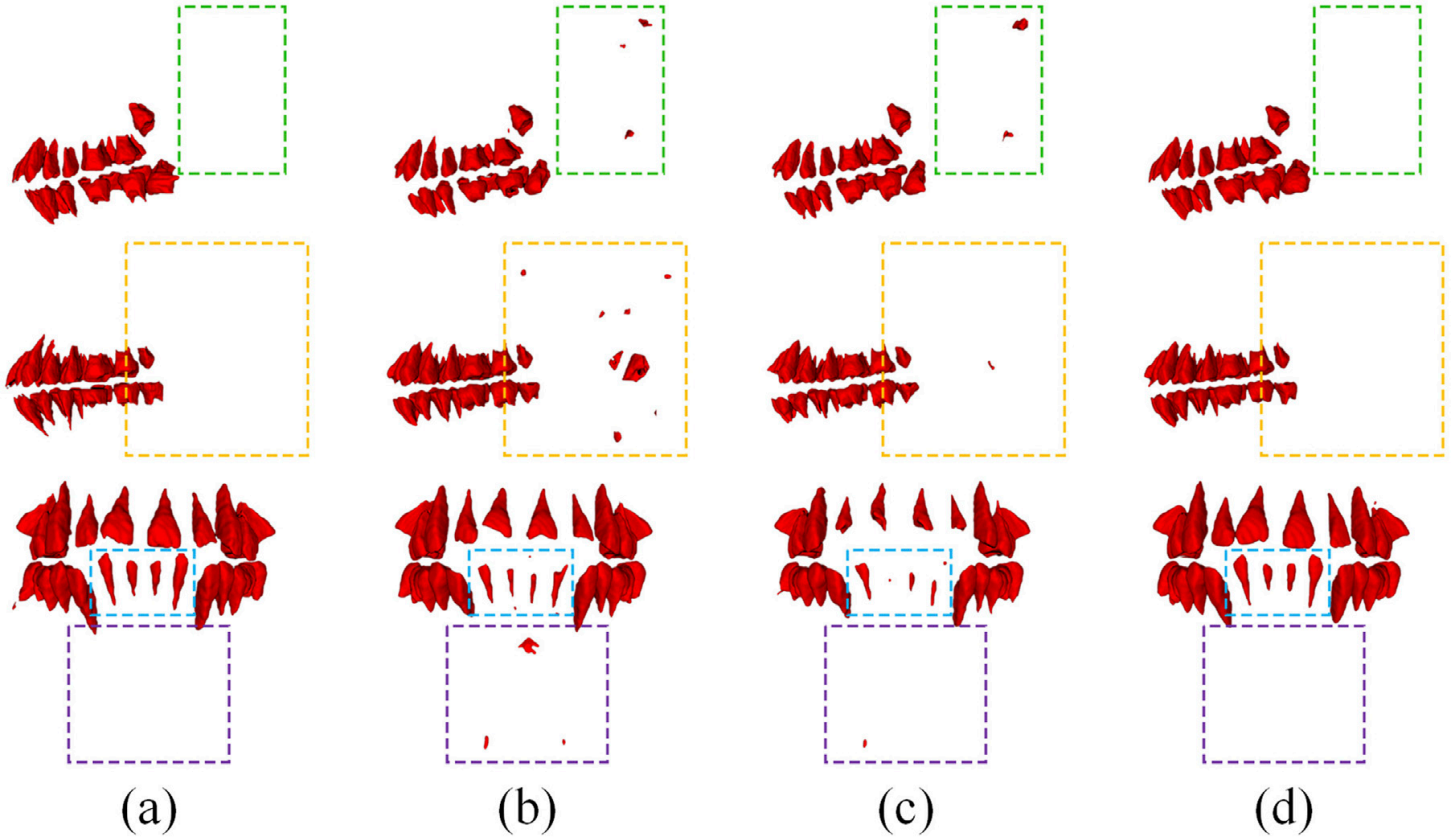
Typical results using different models in tooth detection. **(a)** Ground Truth, **(b)** CTDC-Net, **(c)** U-Net, **(d)** Attention U-Net.

Table 1 presents a quantitative comparison between Attention U-Net and other networks. As shown, Attention U-Net outperforms CTDC-Net and U-Net in terms of HD and ASD metrics. The average CD between the centroids of the tooth descriptors and the true centroids, obtained from the results of Attention U-Net, is reported as 2.63 ± 1.26 voxels, with the mean and standard deviation within the perturbation range. Figure 2 compares the actual detection results of the networks. It can be observed that only the Attention U-Net, which focuses on the region of interest more, avoids predicting non-tooth regions as teeth, while U-Net even faces the possibility of missing teeth.

**TABLE 1:** Comparison the results of tooth detection.

Methods	HD (mm)	ASD (mm)	CD (voxel)
CTDC-Net Gong et al. (2024)	137.71 ± 15.35	4.28 ± 2.76	—
U-Net	83.72 ± 52.11	1.06 ± 0.77	—
Attention U-Net	10.46 ± 2.38	0.51 ± 0.03	2.63 ± 1.26

### 3.4 Tooth segmentation

#### 3.4.1 Comparison

We compare the proposed ensemble learning method (AU-V-Net EL) with several popular deep learning models, and the comparison results presented in Table 2. Where “P-free” denotes training with centroid-perturbed-free data and “P” indicates training with centroid-perturbed data. Where “Time” is the average time to segment a tooth (a ROI). For V-Net and Attention U-Net, training with perturbed data yields better performance on the test set. Therefore, the AU-V-Net EL was trained only with centroid-perturbed data. As illustrated in Table 2, AU-V-Net EL surpasses all other models across all metrics and exhibits superior robustness, albeit with a significantly longer inference time. In this paper, we argue that SWIN-UNetR’s lower performance than V-Net and Attention U-Net may be because SWIN-UNetR’s windowed attention mechanism relies on larger input sizes to model long-range dependencies. However, the clipped ROI narrows down the contextual scope, which limits its advantages.

**TABLE 2:** Comparison of tooth segmentation results.

Methods	Dataset	Dice (%)	Jaccard (%)	HD (mm)	ASD (mm)	Time (s)
U-Net	P-free	94.54 ± 1.90	89.69 ± 3.09	3.71 ± 2.43	0.39 ± 0.41	0.28
	P	94.41 ± 1.95	89.63 ± 3.16	3.73 ± 2.61	0.411 ± 0.34	0.27
U-NetR	P-free	94.22 ± 3.17	89.22 ± 4.86	5.44 ± 3.20	0.49 ± 0.42	0.29
	P	93.63 ± 4.56	88.29 ± 6.29	5.81 ± 3.47	0.57 ± 0.64	0.29
Swin-UNetR	P-free	95.29 ± 2.00	91.07 ± 3.32	3.87 ± 2.48	0.34 ± 0.28	0.31
	P	94.86 ± 3.14	90.36 ± 4.59	3.88 ± 2.97	0.38 ± 0.60	0.28
V-Net	P-free	95.29 ± 1.39	91.04 ± 2.50	3.41 ± 1.88	0.31 ± 0.11	0.29
	P	95.64 ± 1.13	91.66 ± 2.04	2.89 ± 1.76	0.29 ± 0.10	0.27
Attention U-Net	P-free	94.30 ± 4.15	89.48 ± 6.77	4.98 ± 4.61	0.60 ± 0.78	0.33
	P	95.46 ± 2.38	91.41 ± 3.94	3.59 ± 3.29	0.40 ± 0.57	0.27
AU-V-NetEL (Ours)	P	96.33 ± 0.89	92.94 ± 1.64	2.04 ± 0.87	0.24 ± 0.06	4.72

**FIGURE 3:**
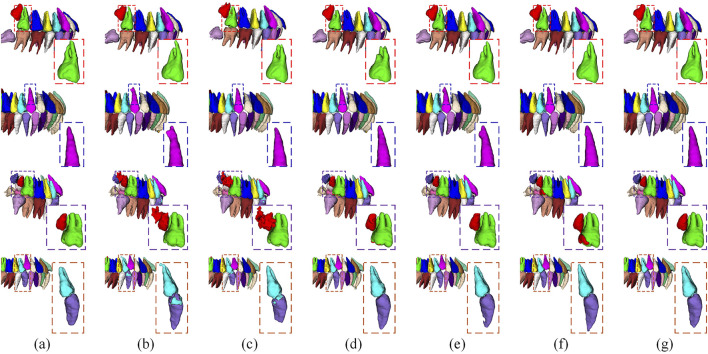
Typical results using different models in tooth segmentation. **(a)** Ground Truth, **(b)** U-Net, **(c)** U-NetR, **(d)** Swin-UNetR, **(e)** V-Net, **(f)** Attention U-Net, **(g)** AU-V-Net EL (ours).

Figure 3 presents a comparison of typical segmentation results across various models. As illustrated in the figure, AU-V-Net EL effectively segments the teeth and successfully captures details that other models either fail to segment or confuse.

#### 3.4.2 Ablation experiment

The ablation experiment primarily analyzes the impact of joint training and varying levels of ensemble training on segmentation performance. Table 3 presents the quantitative results for Dice, Jaccard, HD, ASD, and the proposed composite metric $C_{\mathrm{Metric}}$ , which is positively correlated with segmentation performance and is calculated from Dice, HD, and ASD. In Table 3, ‘V-Net’ and ‘Attention U-Net’ refer to once individual random training, while ‘Joint V-Net’ and ‘Joint Attention U-Net’ refer to once joint training of V-Net and Attention U-Net, respectively. ‘STV’ and ‘STAU’ represent ensembles of 10 times individual trainings of V-Net and Attention U-Net, respectively, while ‘JTV’ and ‘JTAU’ represent ensembles of 10 times joint trainings. ‘AU-V-Net EL’ is the Ensemble of STV, STAU, JTV, and JTAU. All ensemble results are obtained based on the $C_{\mathrm{Metric}}$ .

**TABLE 3:** Comparison of Ablation experiment.

Methods	Dataset	Dice (%)	Jaccard (%)	HD (mm)	ASD (mm)	$C_{\mathrm{Metric}}$
V-Net	P-free	95.29 ± 1.39	91.04 ± 2.50	3.41 ± 1.88	0.31 ± 0.11	1.67 ± 0.25
	P	95.64 ± 1.13	91.66 ± 2.04	2.89 ± 1.76	0.29 ± 0.10	1.75 ± 0.24
Attention U-Net	P-free	94.30 ± 4.15	89.48 ± 6.77	4.98 ± 4.61	0.60 ± 0.78	1.63 ± 0.38
	P	95.46 ± 2.38	91.41 ± 3.94	3.59 ± 3.29	0.40 ± 0.57	1.72 ± 0.30
Joint V-Net	P-free	95.13 ± 1.51	90.75 ± 2.68	3.33 ± 1.83	0.33 ± 0.12	1.66 ± 0.24
	P	95.68 ± 1.13	91.74 ± 2.06	2.99 ± 1.37	0.29 ± 0.08	1.73 ± 0.23
Joint Attention U-Net	P-free	94.53 ± 3.69	89.84 ± 6.14	4.66 ± 4.37	0.54 ± 0.68	1.66 ± 0.38
	P	95.55 ± 1.78	91.53 ± 3.00	3.23 ± 2.48	0.32 ± 0.34	1.73 ± 0.25
STV	P	95.87 ± 0.96	92.09 ± 1.76	2.13 ± 0.86	0.27 ± 0.06	1.88 ± 0.24
STAU	P	96.17 ± 0.91	92.64 ± 1.68	2.09 ± 0.95	0.26 ± 0.06	1.92 ± 0.25
JTV	P	96.03 ± 0.92	92.37 ± 1.69	2.13 ± 0.94	0.27 ± 0.06	1.90 ± 0.26
JTAU	P	96.16 ± 0.96	92.63 ± 1.76	2.08 ± 0.89	0.25 ± 0.06	1.93 ± 0.26
AU-V-Net EL (Ours)	P	96.33 ± 0.89	92.94 ± 1.64	2.04 ± 0.87	0.24 ± 0.06	1.96 ± 0.26

From Table 3, we observe that: (i) Training with centroid-perturbed data outperforms training with centroid-perturbed-free data, regardless of whether single or joint training is employed; (ii) In single training, Attention U-Net performs worse than V-Net, but in the ensemble of 10 times trainings, Attention U-Net surpasses V-Net, underscoring the significance of ensemble learning for enhancing robustness; (iii) The results achieved through ensemble learning are markedly superior to those obtained from single training; (iv) AU-V-Net EL outperforms all other methods across all evaluated metrics.

### 3.5 Tooth position correction

**FIGURE 4:**
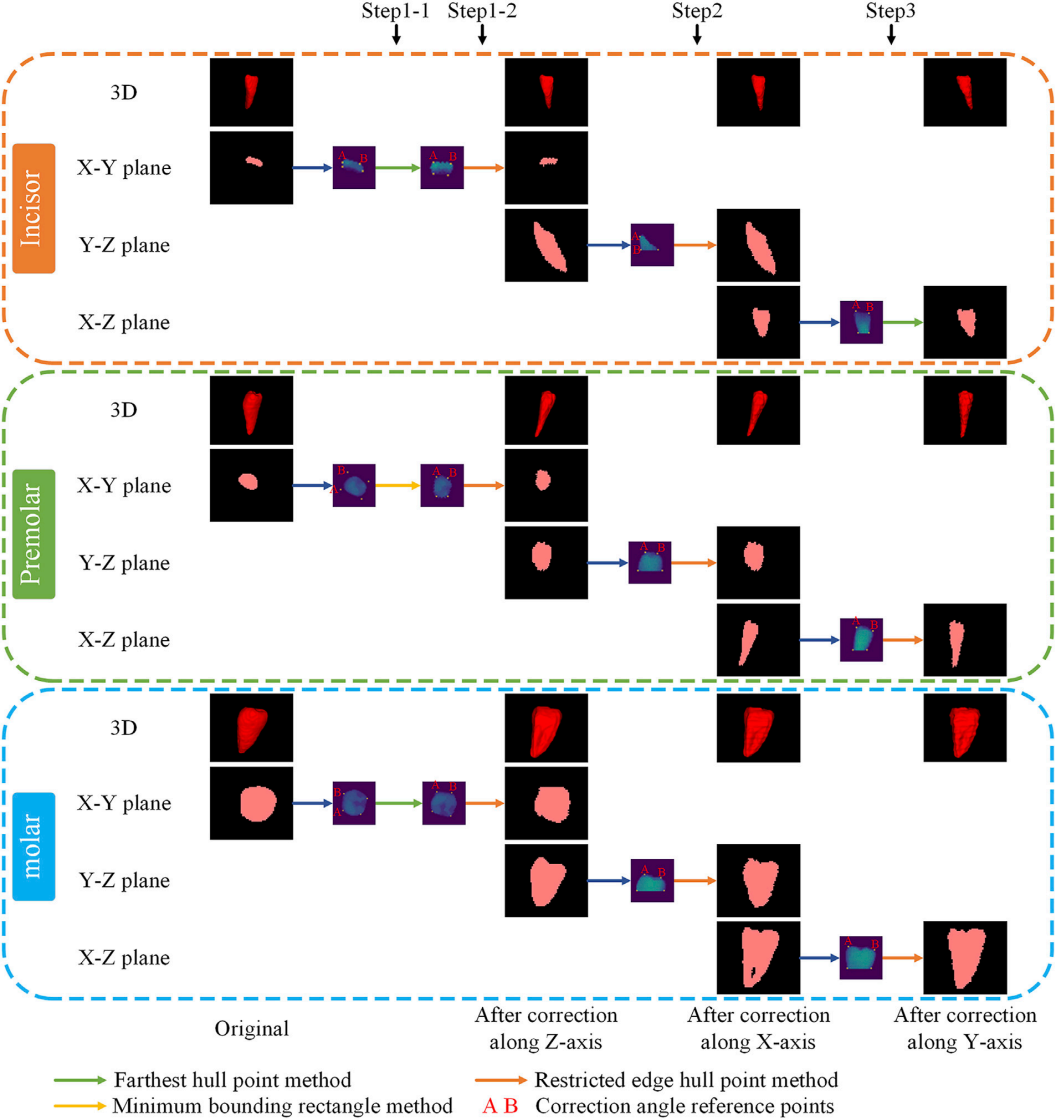
Typical example of positional correction.

Figure 4 illustrates the correction process for three distinct types of teeth: the lateral incisor, the second premolar, and the second M. The small purple images indicate the selected projections and reference points for correction angles obtained through different methods, where points A and B serving as the references for the correction angle.

### 3.6 Root canal working length and angle measurement

#### 3.6.1 Root canal working length measurement

There is no gold standard for validation of root canal work length and angle measurements because all data in this study were collected from CBCT data of living human beings. Therefore, we used the manual measurements of six professional medical students as a reference standard. This study make a comparison of manual and automatic measurement results across nine test cases, encompassing a total of 275 teeth and 350 root canals. Table 4 presents several measurement results.

**TABLE 4:** Root canal measurement results for diverse tooth types.

Tooth type	Auto (mm)	Mean (mm)	M1 (mm)	M2 (mm)	M3 (mm)	M4 (mm)	M5 (mm)	M6 (mm)
Incisors	20.84	20.79	20.56	20.94	21.1	20.62	20.65	20.89
	19.76	19.87	19.92	19.94	19.84	19.67	19.87	19.99
	17.6	17.66	17.85	17.94	17.83	17.42	17.32	17.58
Canines	24.44	24.52	24.19	24.74	24.69	24.65	24.43	24.39
	22.27	22.56	22.63	22.69	22.98	22.68	22.16	22.19
	24.32	24.52	24.59	24.7	24.74	24.54	24.07	24.48
Premolars	18.57	18.6	18.4	18.57	18.54	18.5	18.79	18.78
	18.11	18.25	18.18	18.25	18.15	18.4	18.01	18.5
	16.66	16.64	16.59	16.83	16.62	16.64	16.81	16.33
Molars	18.59	18.62	18.59	18.65	18.65	18.47	18.72	18.62
	16.88	16.85	16.96	16.68	16.97	16.72	16.79	16.96
	18.79	18.85	18.82	18.72	18.72	19.07	18.71	19.05

Statistical findings based on these measurements indicate that the average standard deviation of manual measurements by different medical students for the same root canal is 0.406 mm, highlighting the inherent variability in manual measurement. The average difference between the mean values of automatic and manual measurements is 0.615mm, with a Relative Error of 3.42% ( < 5%), indicating high accuracy and reliability of the automatic measurements.

According to clinicians, the commonly used root canal instruments are available in four lengths: 21 mm, 25 mm, 28 mm, and 31 mm. Therefore, the error in our automatic measurements does not affect instrument selection, can provide valuable clinical guidance. Besides, the manual measurement process is time-consuming, with automated measurements of a tooth averaging only 1.73 s, compared to 27.48 s for manual measurements.

#### 3.6.2 Curvature angle measurement

To address the insufficient spatial resolution of the original dataset for comprehensive pulp chamber visualization, we supplemented the study with high-resolution CBCT scans (0.125 × 0.125 × 0.125 mm^3^) obtained from hospital. The five CBCT cases comprised partial dentition scans, encompassing 17 teeth and 21 root canals. The results were measured manually by a specialized physician, serving as the standard for evaluation.

Root canal curvature angles were categorized into three treatment difficulty grades per established criteria: Grade 1 (0°–10°), Grade 2 (10°–25°), and Grade 3 (>25°). The automated measurements showed a mean angular discrepancy of 2.85° compared to manual references, with two grading misclassifications among 21 root canals (agreement rate: 90.48%).

#### 3.6.3 Length-angle visualization

In this study, after measuring the working length of the root canal, the corresponding voxels for the root in the image are assigned a value calculated as the root canal length multiplied by 100 (for instance, a measurement of 16.95 mm would be represented as 1,695). A circular point is marked at the root apex to denote the root canal curvature angle (for example, a curvature angle of 25° would be displayed as 25). To visualize and facilitate accurate correspondence between root canals and measurements. Furthermore, this study automatically generates an Excel file to document the length and angle of each root canal, highlighting those with a working length greater than 25 mm or less than 15 mm.

## 4 Discussion

This study aims to automatically segment a single tooth from CBCT images and to measure the root canal working length and angle, thereby assisting dentists in preoperative planning. The research is organized into four stages: 1) tooth detection, 2) tooth segmentation, 3) tooth position correction, and 4) measurement of root canal working length and angle.

In the tooth detection stage, we locate the teeth by identifying tooth descriptors. The size of the tooth descriptor is crucial; if too large, neighboring upper and lower tooth descriptors may merge, while if too small, some teeth may be missed. Therefore, future work should consider selecting appropriate descriptor sizes based on tooth type, e.g., larger descriptors for molars and smaller ones for incisors. Although our current dataset does not occur cases of misidentifying non-tooth areas as tooth descriptors, the sample size is too small to ensure consistent performance of the Attention U-Net across all data. Future research could consider initially detecting the entire tooth region and subsequently cropping out only the tooth portion for the next stage of tooth descriptor identification. However, due to variations in individual maxillofacial anatomy and CBCT imaging position, different images may require cropping at different sizes.

In the tooth segmentation stage, our proposed ensemble deep learning method outperforms popular medical image segmentation approaches, demonstrating better performance on unseen test datasets. This method combines the advantages of V-Net and Attention U-Net, and the ensemble of multiple training results mitigates the randomness of individual networks, offering improved robustness. However, the requirement to load and validate multiple models results in longer inference times and increased computational costs, with an average segmentation duration of 4.72 s per tooth. The extended inference time can diminish the clinical application’s convenience and the model’s practical usability. Thus, identifying a suitable model ensemble ratio is crucial. Future research should focus on either retaining only the top-performing models for ensemble learning or selecting the number of models in ensemble learning based on specific requirements. This approach aims to enhance segmentation performance within the desired scope while minimizing inference time to the greatest extent possible.

**FIGURE 5:**
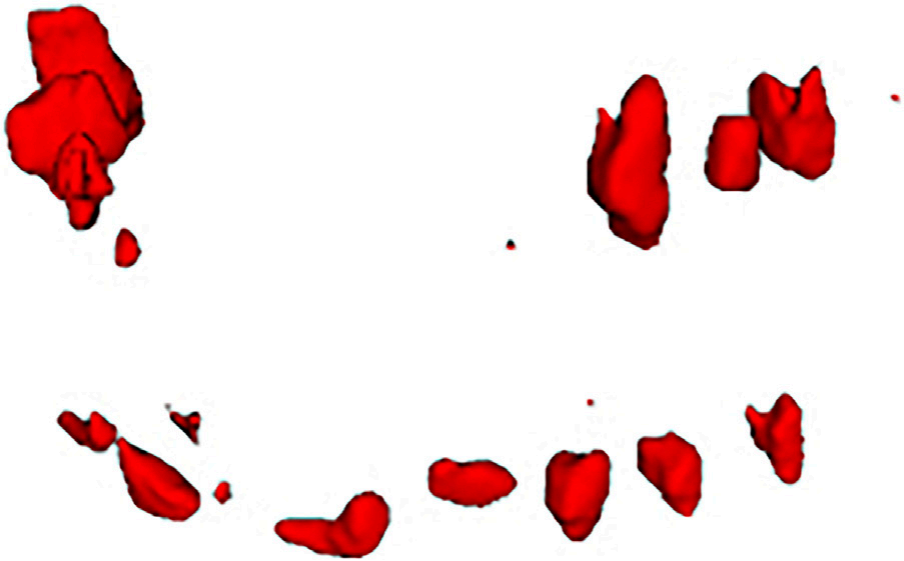
External dataset tooth detection validation results.

**FIGURE 6:**
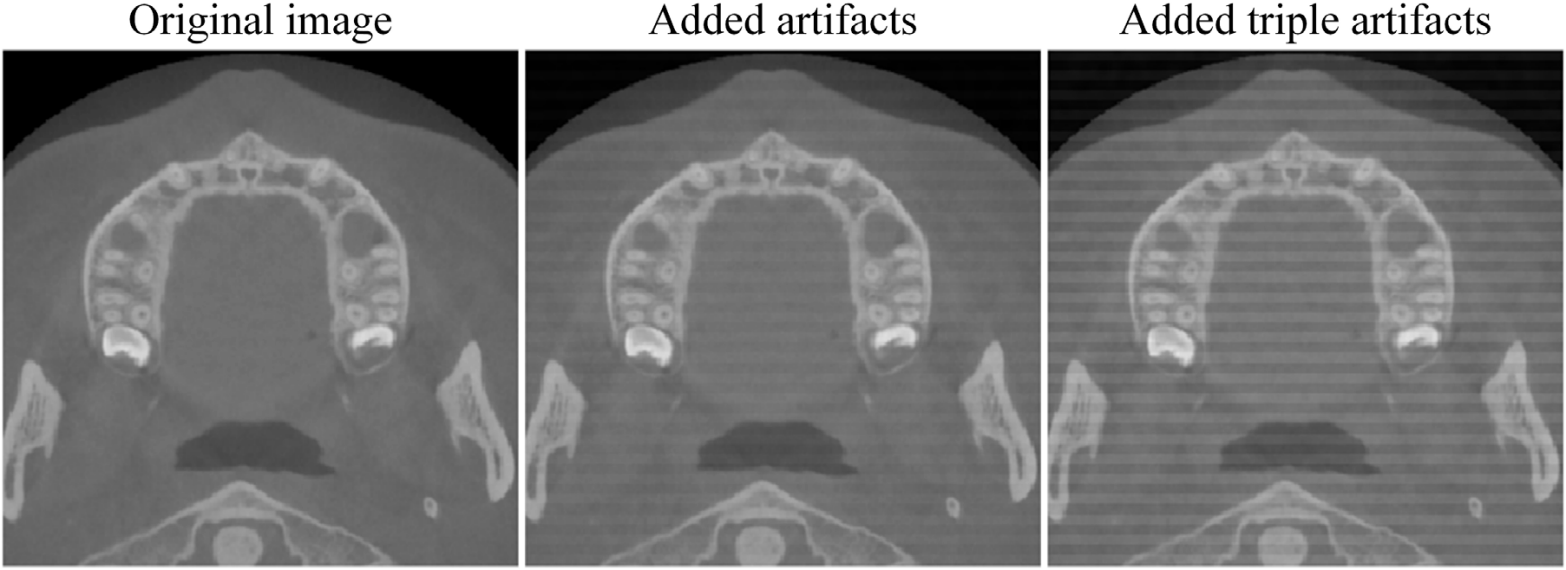
Add artifacts.

In this study, we obtained an external dataset of 20 dental CBCT images Li (2024) to evaluate the performance of our tooth detection model. After adjusting the resolution and dimensions of these images, we applied our tooth detection model and found it failed to accurately locate teeth due to significant dataset differences. The identification result is presented in Figure 5. Subsequently, we added Gaussian and stripe artifacts to the original images (see Figure 6) and retested the model. Under mild artifacts, all nine cases were accurately localized without false positives or negatives, showing no significant difference from the original images. Under heavy artifacts, one case had a non - tooth area misidentified. This indicates the model has some robustness to artifacts, but also highlights its limitations with external data. This underscores the need for adequate training data to enhance the model’s generalizability.

The dataset of this study is limited, comprising only 39 CBCT cases obtained from a single hospital. This insufficient data increases the risk of model overfitting and limits the generalizability of the findings, thereby restricting the wide applicability of potential clinical applications. Future work will focus on training and validating the model on a larger and more diverse dataset to enhance its robustness and generalization capability. This study’s method shows good segmentation results on a small dataset. When trained on larger and more diverse datasets in the future, the advantages of deep learning will be more evident, leading to better segmentation. However, this method requires training multiple models, and cropping ROIs significantly increases data volume, thus raising training time and hardware requirements. In subsequent training of ROI models on larger datasets, we will retain only a portion of the data for training. It aims to preserve data diversity and segmentation performance while minimizing training time.

In the root canal working length measurement stage, the relative error between our automatic measurements and manual measurements is 3.42% ( < 5%), indicating a high level of accuracy in the measurement results. This provides clinicians with an objective and efficient measurement method that yields results consistent with physician estimates, aiding in the root canal diagnosis grading and the selection of instruments and materials in the root treatment. The visualized results enable clinicians to intuitively and accurately correlate the measurements with the root canal, thereby reducing errors in root canal assessment. Additionally, our position correction method minimizes the impact of position errors on the measurements. Currently, there is a lack of research on automatic root canal measurement, and our study contributes to enriching this area of research.

In the root canal curvature angle measurement stage. A significant challenge in this process is the automatic identification of the curvature initiation point. In this study, we identified the root canal curvature initiation point by analyzing the degree of positional change in the center point of each sequential slice layer. The mean difference between the manual and automatic measurement results was 2.85°, with a treatment difficulty factor grading accuracy of 90.48%. However, due to the limited data, the statistical significance was low. Therefore, more high-resolution CBCT images will be acquired in the future to study root canal curvature angles. The method proposed in this study extends the root canal curvature measurement from 2D to 3D space and provides a new method for automatic root canal curvature measurement.

In this study, all images were scanned from human bodies rather than from extracted teeth. Consequently, we were unable to obtain ground truth data through vernier caliper measurements and had to rely on manual measurements conducted by medical professionals as the reference standard, which may not be sufficiently accurate. Future research could utilize extracted teeth as specimens to further validate the effectiveness of the proposed measurement methods.

While our study successfully achieves automatic tooth segmentation, correction, and root canal measurement, these processes are currently performed in stages rather than being fully automated. In the future, we aim to integrate these steps into a unified system for end-to-end automation. Furthermore, developing a network capable of single-stage, end-to-end segmentation of individual teeth is another potential research direction.

## 5 Conclusion

This study proposes a automated method for single-tooth segmentation and root canal measurement, achieving accurate results.

(1) An ensemble deep learning approach for tooth segmentation is proposed, ensembling Attention U-Net and V-Net results using the proposed composite metric, which enhances robustness. The results outperform current methods, with a Dice coefficient of 96.33%, Jaccard coefficient of 92.94%, HD of 2.04mm, and ASD of 0.24 mm.

(2) A root canal automatic measurement method based on connected component analysis is proposed. The relative error for root canal working length measurement between automatic measurement and manual measurement is 3.42% ( <5 %), providing an objective and efficient measurement method that yields results consistent with physician estimates. The results derived from this method provide valuable references for clinical applications in root canal diagnosis grading, instrument selection, and preoperative planning.

(3) An automatic tooth position correction method is developed to improve measurement accuracy and facilitate observation by clinicians. The effectiveness of this correction method was validated by professional doctors.

## Data Availability

The raw data supporting the conclusions of this article will be made available by the authors, without undue reservation.
